# Feasibility of preoperative patient self-assessed frailty: a single-centre pilot study

**DOI:** 10.1016/j.bjao.2026.100539

**Published:** 2026-02-27

**Authors:** James Durrand, Christopher Taylor, Andrew D. Kane, Kerry Colling, Mohammad Sayari, Jochen Einbeck, Nathan Griffiths, Joshua Craig, Lauren Simpson, Gerard Danjoux

**Affiliations:** 1 Department of Anaesthesia and Perioperative Medicine, South Tees Hospitals NHS Foundation Trust, Middlesbrough, UK; 2 Centre for Clinical Research and Improvement, Royal College of Anaesthetists, London, UK; 3 School of Anaesthesia, Health Education England North East, Newcastle, UK; 4 North Yorkshire Academic Alliance of Perioperative Medicine, Middlesbrough and York, UK; 5 Hull York Medical School, University of York, Heslington, York, UK; 6 Department of Mathematical Sciences, Durham University, Durham, UK; 7 School of Anaesthesia, Health Education England North West, Manchester, UK

**Keywords:** frailty, perioperative medicine, perioperative outcomes, preoperative assessment, self-assessment, shared decision-making

## Abstract

**Background:**

Frail patients are at risk of poorer perioperative outcomes, with early identification facilitating shared decision-making and care planning. Preoperative self-assessment is understudied and may support early screening. We developed and piloted a modified Rockwood Clinical Frailty Scale (CFS) using lay language for patient self-assessment before major surgery. Our aim was to pilot the feasibility of preoperative patient self-assessment of frailty using the modified tool. Agreement with a clinician CFS assessment was evaluated.

**Methods:**

We recruited 80 patients out of those aged ≥65 yr attending for preoperative assessment at a single centre in the UK. Participants self-assessed using the modified CFS, with a clinician blinded to this completing a parallel standard CFS assessment. Agreement was evaluated using Cohen's kappa and a linear mixed-effects model, treating the CFS as both a 9-point scale and as three categories. A *post hoc* subgroup analysis was undertaken in participants aged ≤74 yr and those aged >74 yr.

**Results:**

Eighty participants were screened to determine interest and then recruited with a median age of 74 yr between April 2023 and March 2024. Seven (8.8%) participants were frail (CFS ≥5). Seventy-three participants (91%) successfully completed the self-assessment. Seventy-seven percent of self-assessments matched the clinician assessment exactly or were within 1 point. Overall agreement was moderate (kappa coefficient=0.433, *P*<0.001). This was stronger when treating the CFS as a 9-point scale and in participants aged ≤74 yr. Where disagreement occurred, patients tended to overestimate their frailty with respect to the clinician (mean difference, 0.263 CFS points; *P*=0.055).

**Discussion:**

This single-centre pilot study evaluated the feasibility of preoperative frailty self-assessment. We report that patients can complete a modified CFS achieving moderate agreement with the clinician’s CFS assessment. This study provides an important foundation for further development and validation work to establish feasibility and suitability to incorporate within early preoperative screening initiatives.

Frailty is a multidimensional clinical syndrome, frequently associated with ageing, resulting in diminished capacity to respond to and recover from physiological stressors including major surgery.^1^^,^^2^ The volume of frailty in the UK surgical population is high, presenting a significant challenge to perioperative services underlined by the findings of SNAP3.^3^^,^^4^ One in five people presenting for surgery are frail, rising to higher than 30% in the >80 age group.^3^ Frail patients face a markedly increased risk of perioperative morbidity, mortality, a prolonged length of hospital stay, hospital readmission, and discharge to a destination other than their own home.^4^, ^5^, ^6^

UK Centre for Perioperative Care (CPOC) guidance advocates early preoperative identification of patients living with frailty to facilitate shared decision-making and perioperative planning.^7^ Several frailty identification tools exist of varying complexity, with the Rockwood Clinical Frailty Scale (CFS) the most widely used and validated in the preoperative setting.^8^^,^^9^ The CFS is a 9-point ordinal scale (1–9) with visual prompts and descriptive text allowing healthcare staff to categorise patients from ‘very fit’ to ‘terminally ill’. The scale also facilitates broader categorisation of patients depending on score: 1–3= not frail; 4= vulnerable/pre-frail; 5–9= frail. Recent work has highlighted the escalation in perioperative risk with a CFS of ≥4.^3^^,^^9^ The CFS demonstrates both accuracy and inter-rater reliability for perioperative frailty assessment^10^^,^^11^; however, it routinely requires in-person evaluation by trained medical or nursing staff.

Preoperative patient self-assessment of frailty may be a valuable option if it can be performed accurately. National initiatives are underway to support earlier remote and digitally facilitated patient screening,^12^ which also aids resource allocation by preoperative services.^13^ A number of frailty assessment tools have shown promise for self-assessment in the community setting^14^^,^^15^; however, we are unaware of any studies evaluating the CFS in preoperative populations.

We developed a modified CFS for patient self-assessment using lay language. Our aim was to pilot preoperative patient self-assessment of frailty using the modified tool. We compared agreement between patient self-assessment using our modified Rockwood CFS to the assessment by a trained clinician using the standard scale; the latter treated as the gold standard.

## Methods

### Ethical approvals

Full approval was obtained from an NHS Research Ethics Committee (REC) (21/NI/0133 HSC REC B). We conducted this pilot study in the South Tees Hospitals NHS Foundation Trust between April 2023 and March 2024.

### Development of a modified Clinical Frailty Scale for patient self-assessment

**Fig 1:**
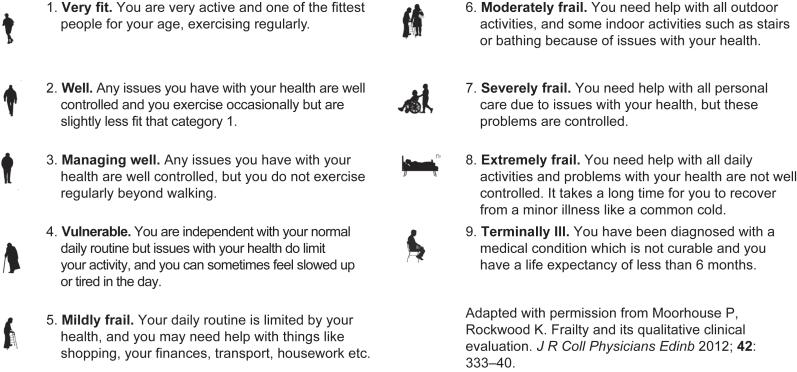
Modified Clinical Frailty Scale (CFS) for patient self-assessment.

A draft modified CFS for patient self-assessment was developed using lay language in place of the standard descriptive text across domains 1–9. Visual aids remained unchanged. This was evaluated by a focus group of five preoperative patients and five clinical staff (two consultant anaesthetists and thee nurses) working in the preoperative assessment clinic (PAC). The modified CFS was further adapted based on feedback. This version was piloted over a 1-week period in the PAC. The final modified tool (Fig. 1) was approved by the study REC.

### Participant recruitment

Patients aged 65 yr and older scheduled to attend the PAC at a single centre (South Tees Hospitals NHS Foundation Trust) before potential major surgery (NICE NG45^16^) were eligible to participate. Patients were excluded if they were unable to provide informed consent or a language barrier prevented completion of the modified CFS.

Eligible patients were screened by telephone to determine initial interest. Interested patients received the study participant information sheet and a copy of the modified CFS for self-assessment by post before their PAC appointment. Written informed consent was completed in person when attending the PAC.

### Procedures

Participants completed their self-assessment using the modified CFS on paper, with a research team member in attendance for support where required. PAC staff were blinded to the self-assessment, with the results placed in a sealed envelope. During the PAC assessment, the attending nurse (trained in CFS evaluation) then completed the routine standard CFS assessment. Results were recorded on the hospital’s electronic preoperative assessment platform (Synopsis IQ, Brookwood, UK). The clinician CFS assessment was retrieved from the Synopsis platform and recorded on the study database by a study investigator after the patient’s appointment. Previous research at our institution has demonstrated substantial agreement between CFS scores completed by preoperative nursing and senior medical staff.^17^ The score completed by the attending PAC nurse was therefore considered the ‘gold standard’ for evaluation.

Additional participant data collected included age, biological sex, ASA physical status classification, surgical specialty, and comorbidity profile. For 33 patients where ASA physical status classification was not recorded in the PAC, this was determined by an independent consultant anaesthetist blinded to the CFS assessments who retrospectively reviewed the relevant preoperative clinical records.

### Statistics

A study sample size of 80 participants was selected in keeping with previously published guidance for pilot and feasibility studies.^18^ The pre-determined statistical analysis plan included descriptive statistics and Cohen’s kappa to assess agreement between clinician assessment and patient self-assessment. Agreement was rated according to a standardised scale (<0, no agreement; 0.00–0.20, slight agreement; 0.21–0.40, fair agreement; 0.41–0.60, moderate agreement; 0.61–0.80, substantial agreement; and 0.81–1.00, almost perfect agreement). Further analyses consisted of determining differences and agreement for the CFS as a 9-point scale and by three-level subcategories: non-frail (CFS 1–3), pre-frail (CFS 4), and frail (CFS 5–9).

A *post hoc* subgroup analysis was undertaken for agreement in patients aged 74 yr and younger and those older than 74 yr of age.^19^ This cut-off was selected based on the cohort median age. To investigate the vertical alignment between self-assessment and clinician assessment (whether the mean scores were on the same level), a linear mixed-effects model was utilised (Supplementary material 1). This analysis also investigated whether the presence of such alignment depends on age, ASA physical status classification, or number of comorbidities.

## Results

### Recruitment and patient characteristics

**Fig 2:**
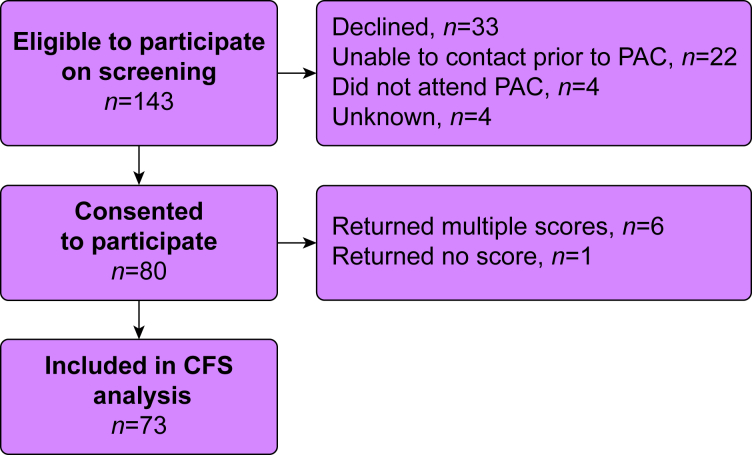
Study participant flow. CFS, Clinical Frailty Scale; PAC, preoperative assessment clinic.

A total of 143 patients met the inclusion criteria on screening and were approached to participate. Eighty participants (55.9%) were recruited and provided written informed consent. Results from seven participants were excluded from the CFS analysis. Figure 2 summarises participant flow through the study including reasons for non-participation and exclusion from the analysis.

Characteristics, surgical specialty, and clinician CFS assessments for the 80 participants are summarised in Table 1. The median (IQR [range]) age was 74 (70–77 [65–87]) yr, with 45 (56%) participants having an ASA physical status score ≥3 and 54 (68%) demonstrating multimorbidity (two or more chronic health conditions). Clinician CFS assessment judged 8.8% of the cohort to be frail (CFS ≥5). The majority of participants were planned for vascular or orthopaedic surgery.

**Table 1:** Characteristics of the study participants (*n*=80). Values are *n* (%) unless indicated. CFS, Clinical Frailty Scale; IQR, interquartile range.

Biological sex (M/F)	50 (62.5)/30 (37.5)
Age, median (IQR [range])	74 (70–77 [65–87])
65–69 yr	19 (23.7)
70–74 yr	26 (32.5)
75–79 yr	21 (26.3)
80–84 yr	10 (12.5)
85–90 yr	4 (5.0)
ASA physical status classification	
1	3 (3.8)
2	32 (40.0)
3	43 (53.8)
4	2 (2.5)
Comorbidities	
Asthma	8 (10)
Chronic obstructive pulmonary disease (COPD)	14 (17.5)
Hypertension	52 (65.0)
Coronary artery disease	21 (26.3)
Heart failure	12 (15.0)
Peripheral vascular disease	22 (27.5)
Anaemia	17 (21.3)
Type 2 diabetes mellitus	13 (16.3)
Chronic kidney disease	9 (11.3)
Transient ischaemic attack or stroke	7 (8.8)
Osteoarthritis	28 (35.0)
Rheumatoid arthritis	1 (1.3)
Anxiety	6 (7.5)
Depression	7 (8.8)
Dementia	1 (1.3)
Surgical specialty	
Orthopaedics	25 (31.3)
Vascular	20 (25.0)
Upper gastrointestinal	11 (13.8)
Urology	10 (12.5)
Colorectal	5 (6.3)
Gynaecology	4 (5.0)
Breast	2 (2.5)
Ear, nose, and throat	1 (1.3)
Maxillofacial	1 (1.3)
Neurosurgery	1 (1.3)
Frailty (clinician CFS assessment)	
Non-frail (CFS 1–3)	49 (61.3)
Pre-frail (CFS 4)	24 (30)
Frail (CFS 5–9)	7 (8.8)

Characteristics of the seven patients who did not complete the self-assessment correctly are included in Supplementary material 2. This group were marginally older (median age, 76 yr) and were all classified as ASA physical status class 3 with one participant assessed as frail and six demonstrating multimorbidity.

### Agreement between clinician assessment and patient self-assessment

Table 2 presents a comparison of clinician CFS assessments and patient CFS self-assessments for the 73 participants included in the analysis. No scores >6 were recorded by clinicians or participants. Patient and clinician assessments were identical in 30 (41%) cases, within 1 point in 26 (36%) cases, and 2 or more points apart in 17 (23%) cases. On the 43 occasions where scores differed, patients assessed themselves as frailer than the clinician assessment 30 times (70%), and less frail 13 times.

**Table 2:**
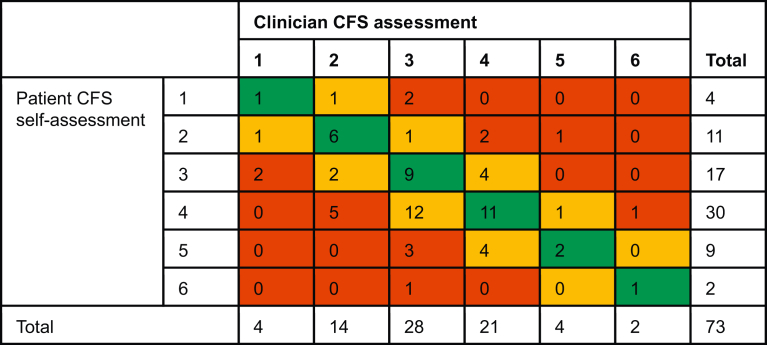
Cross-tabulation of agreement between patient CFS self-assessment and clinician CFS assessment. Green indicates perfect agreement; amber indicates difference in CFS=1, and red indicates difference in CFS ≥2 apart. CFS, Clinical Frailty Scale.

Six patients from this cohort were categorised as ‘frail’ (CFS ≥5) by a clinician. Participants agreed in three of those cases and self-scored as not frail in three. Of those three participants, two self-assessed as pre-frail (CFS 4) against clinician CFS of 5 and 6. One participant self-assessed as CFS 2 (non-frail) against a clinician CFS of 5 (frail).

For the cohort overall, participant self-assessment and clinician assessment demonstrated moderate agreement (kappa coefficient= 0.433, *P*<0.001). Agreement was weaker when the CFS was evaluated as three subcategories (non-frail, pre-frail, frail) rather than the standard 9-point scale (kappa coefficient= 0.319, *P*= 0.003).

The estimate of score difference determined by the linear mixed-effects model suggested the participant self-assessment was 0.263 (*P*= 0.055) CFS points higher than the clinician score overall.

### Subgroup analysis

In a subgroup analysis, participants ≤74 yr of age demonstrated the greatest agreement (moderate, kappa coefficient= 0.485, *P*<0.001), whereas the agreement was only fair in participants aged >74 yr (kappa coefficient= 0.351, *P*= 0.048). As presented in Table 3, this reflects the higher number of exact matches in those ≤74 yr. However, where the scores did not match, participants ≤74 yr tended to self-assess with a higher CFS than the clinician. In contrast, for patients >74 yr, there were fewer absolute matches, but any non-matches were symmetrically distributed above and below the clinician CFS assessment. These insights are corroborated by the mixed-effects analysis (Supplementary material 3), which shows that for the >74 age group, the estimated mean difference between the two scores is minor (0.014 CFS points, *P*= 0.945), whereas for the ≤74 age group, the estimated patient score is 0.475 CFS points higher than the clinician assessment (*P*= 0.008). A similar analysis using ASA physical status scores and number of comorbidities showed that these variables did not impact vertical alignment between participant self-assessed and clinician assessed scores (Supplementary material 3). Separate weak to moderate correlations of frailty with age, ASA physical status class, and comorbidities are also included in Supplementary material 4.

**Table 3:**
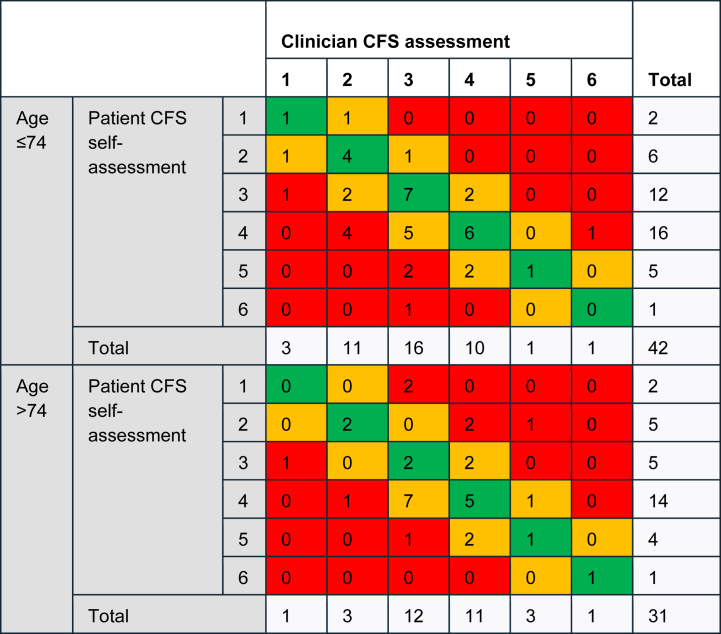
Cross-tabulation of agreement between patient CFS self-assessment and clinician CFS assessment in patients aged ≤74 and >74 yr. Green indicates perfect agreement, amber indicates difference in CFS=1, and red indicates difference in CFS ≥2 apart. CFS, Clinical Frailty Scale.

## Discussion

This single-centre pilot study demonstrates that most patients can complete our modified CFS for self-assessment (using lay language) within a preassessment process for major surgery; 91.2% of participants completed the self-assessment correctly. Our cohort demonstrates substantial comorbidity and multimorbidity with more than half rated as ASA physical status class ≥3, in keeping with other recently published studies.^3^^,^^20^ To our knowledge, this is one of the first studies of self-assessment in a perioperative population, an approach previously shown to be feasible in community-dwelling older adults.^2^^,^^14^

We observed moderate agreement between clinician standard CFS assessment and participant modified CFS self-assessment. This was not affected by increasing ASA physical status class or degree of multimorbidity. Agreement was greatest when considering the CFS in a standard 9-point format rather than as three subcategories, supporting the concept of frailty as a clinical spectrum rather than a threshold. Mean differences between the clinician and participants across the cohort were less than a single point on the CFS, and in 77% of cases, assessments were identical or within 1 point. Although a single-point difference is less likely to be clinically significant, 17 participants (23.3%) were 2 points or greater apart. Crucially, the study captured only six participants identified as ‘truly frail’ on clinician assessment, whereas half agreed with the clinician, half self-assessed as either ‘pre-frail’ or ‘not frail’. This highlights the need for essential further work to validate the tool for screening purposes.

Overall, where disagreement occurred, participants tended to overestimate their frailty with respect to the clinician. Although the clinician judgement was treated as the gold standard, nine participants considered themselves pre-frail or frail where the clinician did not. Although clinicians have the advantage of a medical record, patients hold unique insights into their daily life, functional state, and independence invisible to the clinician. The patient perspective may be a valuable and currently overlooked adjunct to clinical assessment that could support frailty detection. In addition, for the patient, this reflective process may provide a useful basis for entering a subsequent shared decision-making consultation.

In the *post hoc* subgroup analysis, patients older than 74 yr demonstrated weaker agreement and tended to both over- and underestimate their frailty. One possible explanation is the influence of cognitive impairment or visual impairment on the ability to self-assess accurately. Although not a component of the frailty phenotype,^21^ these contribute to frailty within the accumulation of deficits model,^22^ are prevalent with advancing age, and are an acknowledged limitation of frailty self-assessment tools in the community setting.^15^ In addition, older participants may have been concerned about receiving a label of frailty and the potential implications for their perioperative care. We would expect more prevalent and more severe frailty in older patients. Although they may be more likely to undergo automatic face-to-face assessment on age criteria alone, a key objective of a larger-scale study would be to establish accuracy in this group.

We acknowledge several important limitations. Firstly, this was a single-centre study including only participants with confidence in written and spoken English. We also collected limited wider patient data and participants were required to express interest during a pre-screening telephone call. These issues limit wider generalisability. Secondly, although our cohort shares many characteristics with other larger recently published studies, our study contains fewer frail patients than previously observed.^3^ This may reflect our selective recruitment of elective surgical cases only. Crucially, half of these participants did not self-assess as frail. In addition, the maximum CFS score included in the analysis was 6, reflecting a lack of more severe frailty within the cohort. Thirdly, although Cohen’s kappa indicated significant agreement, this was assessed against a null hypothesis where *κ*= 0 (agreement equal to what would be expected purely by chance). This may not be an ideal null hypothesis, because even a poorly performing instrument can produce kappa values slightly above zero.^23^ Testing a null hypothesis that Cohen's kappa returns not more than ‘slight’ agreement (*κ*= 0.2) against the lowest possible target value corresponding to ‘moderate’ agreement (*κ*= 0.41, achievable according to this study) would require a sample size of 105 participants. Finally, our blinding process prevented exploration of why, despite previous development work, 8.8% of participants were unable to complete the self-assessment tool. Although the number of patients in this group was small, all were ASA physical status class 3, the majority demonstrated multimorbidity, and a single additional patient was judged by the clinician as frail. In addition, the scope of the study did not allow formal assessment of cognitive status. Most errors were for double-scoring, and although an electronic questionnaire would largely negate this, further targeted qualitative work is necessary to fully understand reasons for non-completion and enhance the user friendliness of the tool critically for the most vulnerable and comorbid patients. We suggest this could be nested within a future multicentre study also recruiting a larger sample size and utilising a targeted sampling approach to ensure both a wider geographical spread of centres and a study cohort demonstrating the full spectrum of the frailty syndrome.

A modified clinical frailty score (CFS) self-assessment has the potential to support preoperative screening and rationalise allocation of finite face-to-face preassessment capacity. This aligns with national initiatives to implement earlier screening with electronically facilitated questionnaires.^13^^,^^24^ This pilot study provides the basis for further work towards establishing full feasibility and a clinically acceptable degree of accuracy for use in this context.

## Authors’ contributions

Conception and design: JD, NG, JC, GD

Acquisition of data: CT, KC, NG, LS

Analysis and interpretation of data: CT, JD, ADK, MS, JE, LS, GD

Drafting and revision of the article for important intellectual content: all authors

Final approval of the version to be published: all authors

Agree to be accountable for all aspects of the work thereby ensuring that questions related to the accuracy or integrity of any part of the work are appropriately investigated and resolved: all authors

## Funding

Tees Valley Research Alliance.

## Declarations of interest

The authors declare that they have no conflicts of interest.
