# Supplementary material for: Feasibility of preoperative patient self-assessed frailty: a single-centre pilot study
Source: BJA Open. 2026 Feb 27;17:100539. doi: 10.1016/j.bjao.2026.100539 (PMC12964273; doi:10.1016/j.bjao.2026.100539)
Supplement: Multimedia component 1 [file mmc1.docx]

**Supplementary File S1.**

**Details of linear mixed-effects model**

To investigate the effects of the covariates on the strength of agreement between scores, a linear mixed-effects model was set up. The linear mixed-effects model allows for the assessment of covariate impacts and interactions when comparing CFS frailty scores. We consider the scores produced by the patients and clinicians as pertaining to assessment groups. We denote by $y_{ij}$ the measured score for patient $i$ on group $j$, and by the vector $x_{i}$ any covariates of interest for patient $i$. Then, a linear mixed-effects model can be formulated as:

$y_{ij}={\gamma1}_{\left\{ group=Patient’s self-score \right\}}+x_{i}^{T}\beta+\mu_{i}+\varepsilon_{ij},$

where the terms involving the $\gamma$ and $\beta$ are fixed effects, and $\mu_{i}$ is a patient-level random intercept. In the summation term, the pre-operative assessment clinical score serves as the reference category. The fixed effect parameter $\gamma$captures the agreement differences of interest. In the results reported in this paper, only one covariate (age, ASA grade, or number of comorbidities) was included at a time, but also considering the addition of interaction terms between the grouping variable and the respective covariate. The linear mixed-effects models were fitted using function *lmer* in R package **lme4**. All the analysis was performed using the R project statistical software.
