# Supplementary material for: Feasibility of preoperative patient self-assessed frailty: a single-centre pilot study
Source: BJA Open. 2026 Feb 27;17:100539. doi: 10.1016/j.bjao.2026.100539 (PMC12964273; doi:10.1016/j.bjao.2026.100539)
Supplement: Supplementary file 2 — Multimedia component 2 [file mmc2.docx]

**Supplementary File S2.**

| **Participant Characteristics (n= 7)** | |
| --- | --- |
| **Biological Sex (M/F)** | 3 / 4 |
| **Median age (IQR [range])** | 76 (71-82 [68-86]) |
| 65 – 69 years | 2 |
| 70 – 74 years | 1 |
| 75 – 79 years | 1 |
| 80 – 84 years | 2 |
| 85 – 90 years | 1 |
| **ASA** | |
| I | 0 |
| II | 0 |
| III | 7 |
| IV | 0 |
| **Co-morbidities** | |
| Asthma | 2 |
| Chronic Obstructive Pulmonary Disease (COPD) | 1 |
| Hypertension | 4 |
| Coronary artery disease | 2 |
| Heart Failure | 0 |
| Peripheral vascular disease | 3 |
| Anaemia | 1 |
| Type 2 diabetes mellitus | 3 |
| Chronic Kidney Disease | 2 |
| Transient Ischaemic Attack or Stroke | 0 |
| Osteoarthritis | 2 |
| Rheumatoid arthritis | 0 |
| Anxiety | 0 |
| Depression | 0 |
| Dementia | 0 |
| **Surgical specialty** | |
| Orthopedics | 2 |
| Vascular | 3 |
| Upper Gastrointestinal | 0 |
| Urology | 0 |
| Colorectal | 0 |
| Gynaecology | 0 |
| Other | 3 |
| **Frailty (clinician CFS assessment)** | |
| Non-frail (CFS 1-3) | 3 |
| Pre-frail (CFS 4) | 3 |
| Frail (CFS 5-9) | 1 |
| **Multimorbidity** | 6 |

**Table S1.** Characteristics of participants who did not correctly complete the Clinical Frailty Score self-assessment. CFS = Clinical Frailty Score, ASA = American Society of Anesthesiologists Physical Status Classification.
