# Supplementary material for: Feasibility of preoperative patient self-assessed frailty: a single-centre pilot study
Source: BJA Open. 2026 Feb 27;17:100539. doi: 10.1016/j.bjao.2026.100539 (PMC12964273; doi:10.1016/j.bjao.2026.100539)
Supplement: Multimedia component 3 [file mmc3.docx]

**Supplementary File S3.**

| Model | Fixed effect | Effect Estimate (β coefficient) | Standard error | t-value | P-value |
| --- | --- | --- | --- | --- | --- |
| CFS score ~ group (patient) + (1\|ID) | (Intercept) | 3.250 | 0.127 | 25.594 | <0.001 |
|  | group (patient) | 0.263 | 0.136 | 1.934 | 0.055 |
| CFS score ~ group (patient) + age≤74 + group (patient): age≤74 + (1\|ID) | (Intercept) | 3.571 | 0.190 | 18.807 | <0.001 |
|  | group (patient) | -0.014 | 0.205 | -0.070 | 0.945 |
|  | Age≤74 | -0.571 | 0.253 | -2.257 | 0.026 |
|  | group (patient):age≤74 | 0.489 | 0.271 | 1.806 | 0.075 |
| CFS score ~ group (patient) + age>74 + group (patient): age>74 + (1\|ID) | (Intercept) | 3.000 | 0.167 | 17.913 | <0.001 |
|  | group (patient) | 0.475 | 0.177 | 2.681 | 0.008 |
|  | age>74 | 0.571 | 0.253 | 2.257 | 0.025 |
|  | group (patient):age>74 | -0.489 | 0.271 | -1.806 | 0.073 |
| CFS score ~ group (patient) + ASA ≥3 + group (patient): ASA ≥3 + (1\|ID) | (Intercept) | 2.943 | 0.187 | 15.753 | < 0.001 |
|  | Group (patient) | 0.229 | 0.199 | 1.146 | 0.256 |
|  | ASA ≥3 | 0.546 | 0.249 | 2.192 | 0.030 |
|  | Group (patient):ASA ≥3 | 0.086 | 0.274 | 0.315 | 0.754 |
| CFS score ~ group (patient) + number of comorbidities + group (patient): number of comorbidities+ (1\|ID) | (Intercept) | 2.604 | 0.192 | 13.587 | < 0.001 |
|  | Group (patient) | 0.338 | 0.222 | 1.520 | 0.133 |
|  | Number of comorbidities | 0.237 | 0.056 | 4.245 | < 0.001 |
|  | Group (patient): Number of comorbidities | -0.029 | 0.065 | -0.446 | 0.657 |

The model involving age is fitted in two different parameterizations for simple access to p-values.

**Table S1.** Results of linear mixed-effects models. CFS = Clinical Frailty Scale, ASA = American Society of Anesthesiologists Physical Status Classification.
