# Supplementary material for: Feasibility of preoperative patient self-assessed frailty: a single-centre pilot study
Source: BJA Open. 2026 Feb 27;17:100539. doi: 10.1016/j.bjao.2026.100539 (PMC12964273; doi:10.1016/j.bjao.2026.100539)
Supplement: Multimedia component 4 [file mmc4.docx]

**Supplementary File S4**

|  | Patient CFS self-assessment | Clinician CFS assessment | Age | Number of comorbidities | ASA |
| --- | --- | --- | --- | --- | --- |
| Patient CFS self-assessment | 1 | .451** | 0.168 | .361** | .296* |
| Clinician CFS assessment | .451** | 1 | .280* | .445** | .258* |
| Age | 0.168 | .280* | 1 | .235* | .269* |
| Number of comorbidities | .361** | .445** | .235* | 1 | .286* |
| ASA | .296* | .258* | .269* | .286* | 1 |

** Correlation is significant at the 0.01 level (2-tailed).

* Correlation is significant at the 0.05 level (2-tailed).

There was a weak positive correlation between age and clinician CFS assessment (r = 0.280, p = 0.012). Similarly, ASA grade showed a weak positive correlation with both patient and clinician CFS assessment. There was a moderate positive correlation between the number of comorbidities and both patient and clinician CFS.

**Table S1.** Correlation between patient and Clinician assessment CFS and linear effects model covariates. CFS = Clinical Frailty Scale, ASA = American Society of Anesthesiologists Physical Status Classification.
